# Repeat Procedures Within 30 days in Patients Stented for Malignant Distal Biliary Strictures: Experience of 508 Patients at a Tertiary Referral Center

**DOI:** 10.4021/gr420w

**Published:** 2012-03-20

**Authors:** Michael F Byrne, Calvin HY Chan, Malcolm S Branch, Paul S Jowell, John Baillie

**Affiliations:** a Division of Gastroenterology, Department of Medicine, Vancouver General Hospital, Vancouver, Canada; b Duke University Hospital, Durham, NC, USA; c Cartaret General Hospital, Morehead City, North Carolina, USA

**Keywords:** ERCP, Bile duct stricture, Cancer

## Abstract

**Background:**

Stent related occlusion and migration remains a problem despite attempts to improve stent design over this time period. Flanged polyethylene plastic stents (FPS) remains the stent of choice in most centers. Early failure of stents placed for malignant extrahepatic biliary strictures (MEBS) has not previously been studied in detail. We set out to determine the incidence and reasons for biliary stent change within 30 days of the index procedure in a large tertiary center population during a period where (FPS) was the sole plastic stent used.

**Methods:**

Retrospective analysis of endoscopic retrograde cholangiography (ERCP) was undertaken in patients who were stented for presumed or known MEBS between 1993 and 2001. Patients who required repeat stenting within 30 days were identified.

**Results:**

All 508 patients were stented for MEBS. 5.7% of patients had a total of 34 repeat stenting procedures within 30 days of the index procedure; 27of 29 index stents were plastic, 2 were self-expandable metal stents (SEMS), 20 (3.9%) patients had stent failure as the reason for a stent exchange (plastic stent occlusion n = 15, mean time to stent change 14 ± 8.3 days; metal stent occlusion n = 2, mean time to stent change 24.5 ± 7.8 days; plastic stent migration n = 3, mean time to stent change 25 ± 5.3 days). There was a statistically significant difference in the time to stent change between the occluded plastic stent and migrated plastic stent cases (P = 0.045, 95% CI -21.7 to -0.29). 6 patients spent at least 2 additional days in hospital as a result of stent failure.

**Conclusions:**

Early stent failure is an uncommon problem, especially in patients with SEMS. Early plastic stent failure appears to occur sooner with stent occlusion than with stent migration. Early stent failure is associated with significant morbidity and bears an economic impact in additional procedures and hospital stay.

## Introduction

Endoscopic biliary stenting is established as the preferred treatment for palliating malignant extrahepatic biliary strictures (MEBS). Studies have confirmed that it is effective in improving quality of life in jaundiced patients in whom surgical cure is not possible, or deemed too high risk, and reduces jaundice pre-operatively in surgical candidates [1-3]. Compared to surgical drainage procedures, endoscopic stent placement for MEBS has a reduced morbidity and results in a shorter hospital stay [1]. Polyethylene stents can occlude or kink, causing stent failure [1, 4-6]. Stent occlusion is felt to be due to clogging with sludge, and bacterial infection may play a part in this [3, 7].

Optimal stent design has essentially remained unchanged in the last 15 years. Attempts to improve plastic stent longevity by eliminating side holes and changing stent material from polyethylene to Teflon (Tannenbaum stent, Cook UK, Letchworth, Hertfordshire) [8], limiting the central lumen by using a star shaped design (Viaduct stent, GI Supply, Camp Hill, Pennsylvania, USA) [9], and adding an anti-reflux valve (Fusion Marathon, Cook Inc, Winston, Salem, NC USA [10] have failed to demonstrate superiority. In addition, plastic stents also occasionally migrate from their original position [11].

Placement of self-expanding metal stents has been increasingly used for palliation of unresectable malignant biliary strictures. They are proven to have longer patency and greater complication free survival [12]. However, they are still prone to occlusion and migration, and can be extremely difficult to reposition once deployed. Importantly, they are probably only cost-effective if patient survival is at least 6 months [5, 13-15]. Despite their prolonged patency, this has not resulted in reduced biliary complications [16]. Pre-operative biliary drainage has been challenged over the last 15years, but results of multiple studies over this time frame have been inconclusive, with differing limitations in the respective studies [17, 18]. As such, the flanged plastic polyethylene biliary stent is still the predominant stent used in the early management of malignant biliary strictures, whether it is for resectable, borderline resectable, or unresectable disease.

Several studies have suggested that the average duration of patency of plastic stents is about 3 - 5 months [3, 4, 19]. However, the early failure of stents placed for palliation of malignant extrahepatic biliary strictures (MEBS) has not previously been studied in detail. We set out to determine the rate of biliary stent failure within 30 days of the index procedure in a large tertiary center population, during a period of time when flanged polyethylene stents (FPS) were predominantly used.

## Patients and Methods

A retrospective analysis was made of all ERCP examinations between July 1993, and November 2001, at Duke University Medical Center, Durham, North Carolina. All procedures were performed by gastroenterologists experienced in ERCP at this tertiary referral center. We sought to determine the number of patients who were stented for presumed or known MEBS. Diagnosis was based on presenting symptoms, clinical features, laboratory investigations, abdominal imaging, including computed tomography (CT) and ultrasound (US), and ERCP findings. In each case, any decision that a tumor was inoperable was made in a multidisciplinary setting. The main outcome of interest in this retrospective study was the number of patients who required repeat stenting within 30 days of the index stent placement. Stent patency represented the time between stent insertion and stent dysfunction. Stent failure was defined by evidence of any one or combination of recurrent or worsening liver function tests and bilirubin, cholangitis, and dilated biliary tree on imaging. Stent occlusion was determined visually at endoscopy. Although occlusion of the visible end of a stent does not necessarily mean that the stent cannot drain through its side-holes, we took visual evidence of stent occlusion as confirmation that the stent was non-functional (in the appropriate clinical context of obvious biliary obstruction). The hospital records of all patients were reviewed to obtain clinical details, and identify any other relevant information, such as number of repeat endoscopic procedures needed in each case, number of hospital admissions as a result of stent failure, reason for stent exchange, and type of stent used on each occasion. Any prolongation of inpatient stay resulting from stent failure was also recorded.

### Statistical analysis

Variables analyzed were patient characteristics (age, gender) and etiology of malignant stricture, and time to stent change. Continuous variables were expressed as mean ± standard deviation (SD). Univariate analysis was assessed using unpaired *t* test for continuous variables only. A P value of less than 0.05 was considered statistically significant.

## Results

During the study period of 8 years and 5 months from 1993 to 2001, a total of 508 patients were stented for proven or presumed MEBS.

Twenty-nine (5.7%) patients had a total of 34 repeat stenting procedures within 30 days of the index procedure; 20 (68.9%) were male. The mean age was 70.3 years (range 51 - 92 years). The etiology of the biliary strictures included pancreatic carcinoma (n = 17), cholangiocarcinoma (n = 5), ampullary carcinoma (n = 2), gallbladder carcinoma (n = 1) and metastatic carcinoma (ovarian n = 1, colorectal n = 4). 27of 29 index stents were plastic, 2 were metal (Covered Wallstent™, Boston Scientific Corporation, Natick, Mass, USA). All plastic stents used during the study period were Cotton-Leung (Amsterdam) or, Cotton-Huibregtse biliary stents (Cook, Inc., Winston-Salem, NC, USA).

**Figure 1:**
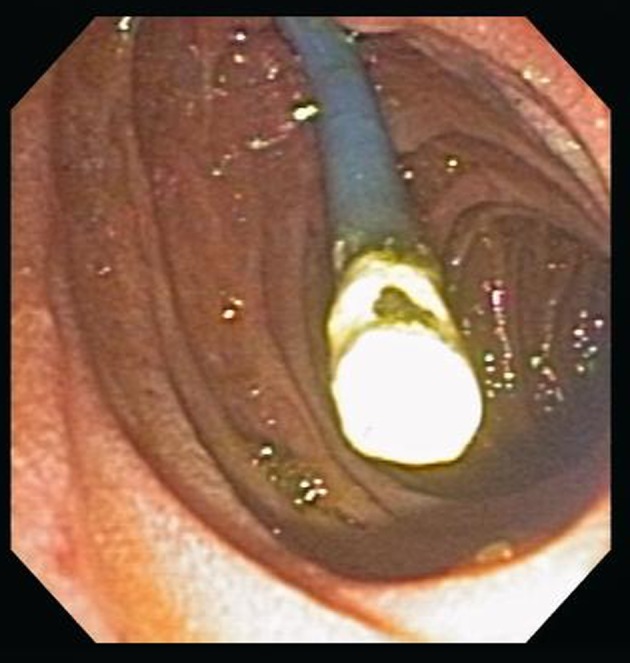
Occluded biliary stent

Of the 29 patients reviewed, 20 (3.9%) had stent related complications leading to a repeat ERCP. 8 (30%) patients were symptomatic (pain n = 5, biliary sepsis n = 3). All had worsening or persistently abnormal liver function tests, 15 of 508 (2.9%) patients with an index plastic stent had stent occlusion as the reason for stent exchange (See Fig. 1). Of these 15 patients, two patients required more than one stent change over the 30 day period (One patient had metastatic colon carcinoma with a total of 3 stent changes due to recurrent occlusion, the other had pancreatic carcinoma requiring 2 stent changes); 4 of 14 occluded plastic index stents were exchanged for expandable metal mesh stents, 3 of 14 occluded index stents were 7 French (Fr) gauge in diameter, one was 11.5 Fr, and ten were 10 Fr. The mean time to first stent change was 14.5 ± 8.4 days.

Three of 508 (0.6%) patients with an index plastic stent had stent migration as the reason for stent exchange. Of these 3 patients, 2 required a second stent exchange, both due to occlusion of the second stent. All three stents were 10 Fr in diameter or greater. The mean time to first stent change was 25 ± 5.3 days, which was significantly longer than in cases with stent occlusion (P = 0.045, 95% CI -21.7 to -0.29).

Nine of 508 (1.8%) patients were scheduled to have an elective stent change within 30 days. Of these 9 patients, 5 (55.6%) were exchanged for a permanent metal stent. Two patients required further diagnostic workup (repeat brushings), and the other two had persistent jaundice, but their stents were found to be patent and in satisfactory position at the time of the second ERCP.

Two occluded metal stents required stent changes at days 4 and 30. One was occluded with debris and clot; the other patient had debris occlusion only. Both were treated with placement of a single plastic stent through the existing metal stent. There was no statistical difference with respect to timing of stent occlusion when metal stent occlusion was compared to that of plastic stents (P = 0.11, 95% CI -23.77 to 2.77) or plastic stent migration (P = 0.94, 95% CI -17.64 to 18.64). Table 1 outlines the characteristics of the patient groups described.

**Table 1:** Patient Characteristics of the Four Groups Requiring Early Stent Exchange

	Occlusion	Migration	Elective	Metal
Total patient (n)	15	3	9	2
Total stent change	18	5	9	2
Mean Age (yrs)	69.3 ± 9.7	78 ± 8.7	70.2 ± 10.5	68 ± 15.6
Gender - male n =	9 (60%)	3 (100%)	7 (77.8%)	1 (50%)
Etiology				
Pancreatic carcinoma	10	1	4	1
Cholangiocarcinoma	2	1	1	0
Ampullary tumor	0	1	1	0
Gallbladder carcinoma	1	0	1	0
Metastatic disease	2	0	2	1
Mean time to stent change	14 ± 8.3	25 ±5.3	15.1 ± 7.7	24.5 ± 7.8

Of the 29 patients who required a stent change within the first 30 days, six (20.7%) spent at least 2 additional days (mean 4.4 days; range 2 - 6 days) in hospital as a result of failure of the index stent.

## Discussion

There are limited data regarding the incidence of early stent failure in malignant biliary strictures. In our study of over 500 patients over an 8 year period, only 20 (3.9%) patients had a repeat stenting procedure within 30 days of the index stent placement as a result of stent failure. To our knowledge, this is the largest such series described in the endoscopic literature. The majority (85%) of stent failure was related to stent occlusion, with only a small proportion (15%) relating to stent migration. In this cohort of patients with early plastic stent failure, we found that stent occlusion occurred earlier than stent migration. Intuitively, stents anchored by a tight stricture should not be inclined to migrate. Consistent with published literature, the vast majority of stents replaced within 30 days of the index procedure were plastic; only 2 were expandable metal mesh stents. Elective repeat ERCP within 30 days was only required in 1.8% of cases, a finding that supports the adequacy of the index ERCP for both diagnostic and therapeutic purposes.

There are several implications of this retrospective study. Early stent failure within a month of the index stent placement exposes these patients to risk of morbidity and mortality, and also impacts on quality of life, as repeated endoscopy is undesirable in the setting of terminal disease. Although early stent failure is a relatively uncommon occurrence, almost 4% of patients in our case series required a second procedure that presumably delayed imminent surgery or hospital discharge. There are also cost considerations: repeat ERCP for stent replacement is expensive, and the complications of stent failure (e.g. cholangitis) incur additional costs, 20% of this group of patients had an additional inpatient hospital stay as a direct result of failure of the index stent, ranging from 2 to 6 days. There are several studies evaluating the use of expandable metal mesh stents in patients with malignant biliary obstruction [15, 19, 20]. Although metal stents are considerably more expensive than plastic ones, it has been shown that metal stents have a longer patency than plastic stents (273 days versus 126 days in one study) [15]. In a study by Menon and colleagues, placement of metal biliary prostheses was shown to represent an effective management strategy for recurrent plastic stent obstruction [20]. The duration of metal stent patency in these patients was shorter than that reported in patients receiving initial metal stent placement, but was still significantly longer than that of the most recently placed plastic stent. The need for fewer repeat procedures may translate into reduced costs in the long term [4, 15, 21]. However, a study by Prat et al found that although the overall cost of initial metal stent placement was $904 less than on-demand exchange of plastic stents and $2127 less than elective exchange of plastic stents, this did not apply to the subgroup of patients who survived less than 3 months [14]. It seems to be generally accepted that patients whose life expectancy is predicted to be 6 months or less are most cost-effectively palliated using plastic biliary stents.

One of the main drawbacks of plastic stents is that they tend to occlude within a few months [3, 4, 19]. In retrospective studies, plastic stent occlusion rates of 10% to 30% after 6 months have been reported [22-24]. The incidence has been found to be as high as 42% in some prospective studies [1, 21, 25]. The mean functional patency of plastic stents from seven randomized trials incorporating a total of 349 patients was 4.9 months [4]. Interestingly, only a small proportion (21%) of occluded stents in this study were the smaller 7 Fr gauge variety, which differs from published literature where occlusion rate is thought to be inversely proportional to stent diameter [24]. Technical considerations, including the diameter of the duodenoscope working channel, preclude placement of significantly larger stents [26]. Plastic stents occlude with sludge and food debris [27]. Mucus glycoprotein and bacteria are believed to play a role in stent occlusion with sludge [28]. Trials of coated stents to reduce the friction coefficient and hence sludge formation or prophylactic antibiotics to delay or prevent stent occlusion have shown conflicting results [28, 29]. The current optimal plastic stent has therefore been unchanged in the last 15 to 20 years.

Plastic stent migration rates of 4% to 8% are commonly reported [11]. In our study, the migration rate was very low in the first 30 days at 0.9%. Migration tended to occur later than occlusion should a plastic stent fail in the first 30 days. This is an expected finding, and supports current practice regarding the timing of elective stent change. At our institution, we perform elective stent changes at 6 months on patients who survive that long to prevent stent complications. However, the majority of patients with malignant biliary strictures succumb to their underlying disease before it becomes necessary to exchange their stents [30].

Limitations in this study include the retrospective nature of the study design. The patient cohort was heterogeneous with multiple etiologies for the malignant biliary obstruction. The lack of a standardized follow-up protocol may have underestimated the number of patients who were asymptomatic during the first 30 days but with biochemical evidence of stent failure. Lack of complete data for the entire ERCP cohort during the study period limited our ability to compare subgroups and identify potential risk factors for early stent failure, although a much larger cohort would have been required to establish statistical significance, given the small numbers in the stent failure group.

In conclusion, 3.9% of patients with malignant biliary obstruction will require a subsequent ERCP within a month of initial biliary stent placement. This is usually related to plastic biliary stent failure. In patients with early plastic biliary stent failure, stent occlusion develops sooner than stent migration. To our knowledge, this is the largest series of early stent failure described so far. Early stent failure has important clinical and economic implications, and impacts negatively on patient quality of life. Further prospective studies assessing characteristics that reduce the need for early repeat biliary intervention would be valuable.
